# Long-term prognostic analysis of children and adolescents with differentiated thyroid carcinoma based on therapeutic response to initial radioiodine therapy

**DOI:** 10.3389/fendo.2023.1217092

**Published:** 2023-08-04

**Authors:** Congcong Wang, Gaixia Lu, Yutian Li, Xinfeng Liu, Guoqiang Wang, Chenghui Lu, Jiao Li, Qiong Luo, Qian Zhang, Ming Sun, Xufu Wang, Renfei Wang

**Affiliations:** ^1^ Department of Nuclear Medicine, The Affiliated Hospital of Qingdao University, Qingdao, Shandong, China; ^2^ Department of Nuclear Medicine, Shanghai Tenth People’s Hospital, Tongji University School of Medicine, Shanghai, China; ^3^ Department of Radiology, Qingdao Women and Children’s Hospital, Qingdao, Shandong, China

**Keywords:** differentiated thyroid carcinoma, children and adolescents, radioiodine therapy, therapeutic response, clinical outcome

## Abstract

**Background:**

The clinical features and prognosis of children and adolescents with differentiated thyroid carcinoma (caDTC) are different from that of adults. Postoperative radioiodine therapy (RIT) was recommended for some intermediate and high risk caDTC patients. The objective of this study was to evaluate the long-term prognosis of pediatric caDTC patients with different responses to initial RIT and to explore the related influencing factors.

**Methods:**

All subjects were assigned to no clinical evidence of disease (NED) group, biochemical persistent disease (BPD) group, or structural/functional persistent disease (S/FPD) group based on the therapeutic response to initial RIT. Then, disease status was evaluated in all three groups at the last follow-up using ATA guidelines. Meanwhile, disease-free survival (DFS) for NED group and the progression-free survival (PFS) for the BPD and S/FPD groups were also assessed.

**Results:**

117 subjects were divided into NED group (n=29), BPD group (n=48) and S/FPD group (n=34) after initial RIT. At the last follow-up, excellent response (ER), indeterminate response (IDR), biochemically incomplete response (BIR) and structurally incomplete response (SIR) rates were 93.10%, 6.90%, 0% and 0% in NED group; 29.17%, 25.00%, 43.75% and 2.08% in BPD group; and 11.77%, 2.94%, 0%, and 85.29% in S/FPD group. The 5-year DFS rate in NED group was 95.5%. The 5-year PFS rates in BPD and S/FPD groups were 79.2% and 48.6%, respectively. For children with structural or functional lesions, longer PFS were found in male children with ^131^I-avid lesions, and post-operative stimulated serum thyroglobulin (sti-Tg) < 149.80 ng/ml.

**Conclusion:**

The response to initial RIT could be helpful for defining subsequent treatment and follow-up strategies for caDTC patients. Post-operative sti-Tg and ^131^I-avidity of lesions are correlated with PFS.

## 1 Introduction

Differentiated thyroid carcinoma (DTC) is a kind of endocrine malignancy, and the incidence of DTC in children and adolescents (caDTC) has increased in recent years (1, 2). Compared with adult patients, caDTC patients tend to have distinctive biologic and clinical features, including larger tumor sizes, more extrathyroidal extension, more cervical lymph node metastases and distant metastases (3–5). However, the mortality of caDTC patients is lower than that of adult DTC patients, despite a higher frequency of recurrent/persistent disease in caDTC patients (2, 6).

“Total or near-total thyroidectomy + selective radioiodine therapy (RIT) + thyroid-stimulating hormone (TSH) suppression therapy” has been recognized as a relatively complete standard therapy model for adult patients with DTC (7, 8). Moreover, evidence-based medical investigations have revealed that RIT plays a significant role in decreasing the risk for disease recurrence and tumor-related death and increasing disease-free survival (DFS) (9, 10). Thus, RIT, as one of the comprehensive therapy strategies for caDTC patients, has gradually been recognized and accepted by doctors around the world (1). The published 2015 American Thyroid Association (ATA) guidelines categorize caDTC patients into three risk groups (including low-risk, intermediate-risk and high-risk) and specifically recommend that post-operative stimulated serum thyroglobulin (sti-Tg) and diagnostic ^131^I whole body scanning (WBS) should be performed first for intermediate- and high-risk patients before deciding whether they should undergo RIT (11). However, the long-term outcome of caDTC patients who underwent RIT, and whether the response to initial RIT could predict long-term outcome need to be further investigated

In this study, we retrospectively analyzed 157 caDTC patients who received RIT from two centers in China, aiming to evaluate the prognostic predictive value of therapeutic response to initial RIT, and to further explore the relationship between clinicopathological characteristics and treatment outcomes.

## 2 Materials and methods

### 2.1 Study conduct

The study protocol was approved by Ethics Committee of the Affiliated Hospital of Qingdao University. Written informed consent to participate in this study was provided by the participants’ legal guardian/next of kin.

### 2.2 Patients

From January 2011 to December 2020, all caDTC patients who underwent RIT in the Affiliated Hospital of Qingdao University and Shanghai Tenth People’s Hospital were reviewed. The inclusion and exclusion criteria for this study are shown in **Table 1** (1–3, 11).

**Table 1:** Inclusion and exclusion criteria in this study.

Inclusion	Exclusion
Underwent total or near-total thyroidectomy	Concomitant malignancy
Histopathological type was diagnosed as DTC	Receipt of other anti-tumor treatments
Intermediate-high recurrence risk	Absence of critical data and follow-up information
Age at the time of initial RIT ≤18 years	
Received at least one standardized RIT and were followed up regularly	
Anti-thyroglobulin antibody (TgAb) negative	

RIT, radioiodine therapy; DTC, differentiated thyroid carcinoma; TgAb, anti-thyroglobulin antibody.

### 2.3 Data collection and RIT procedures

The clinical and pathological data included age at first RIT, sex, histological type, primary tumor size, soft tissue invasion, T stage, N stage, recurrence risk, post-operative sti-Tg, cumulative RIT times and ^131^I activity, which were derived from electronic medical records. Among these, T and N stage were evaluated according to the TNM staging system (8th edition) (9). The recurrence risk of individuals was categorized based on the ATA pediatric risk stratification system (version 2015) (11).

Before RIT, all patients had undergone a low-iodine diet for at least 2 weeks and levothyroxine withdrawal to ensure TSH exceeded 30 μIU/mL. TSH, sti-Tg, TgAb, neck ultrasonography and chest computed tomography (CT) were used as routine evaluation items before RIT. Additionally, magnetic resonance imaging (MRI), ^99m^Tc-methylene diphosphonate single-photon emission computed tomography/computed tomography (^99m^Tc-MDP SPECT/CT), and ^18^F-fluorodeoxyglucose positron emission tomography/computed tomography (^18^F-FDG PET/CT) were performed in selected subjects when metastasis was suspected (9). For caDTC patients, ^131^I activity was administered on a body weight basis (1.0-1.5 mCi/kg). A fixed ^131^I activity of 100-200 mCi (100-150 mCi for residual, recurrent, or lymph node metastasis; 150-200 mCi for distant metastasis) was performed in patients older than 18 years who underwent repeated RIT. Afterwards, post-therapy ^131^I WBS and SPECT/CT were performed 3-7 days after RIT. All patients were administered levothyroxine after RIT for TSH suppression. The therapeutic response to initial RIT was evaluated after 6-12 months with serological examinations (sti-Tg in selected cases, or suppressed thyroglobulin (sup-Tg), and TgAb levels) and with several imaging studies, including neck ultrasonography, diagnostic ^131^I WBS, or other as appropriate. Repeated RIT was conducted when ^131^I-avid lesions were found on the latest post-therapy ^131^I WBS and/or when biochemical remission was confirmed.

### 2.4 Definitions of clinical outcomes

According to the therapeutic response to initial RIT, subjects were assigned to no clinical evidence of disease (NED) group, biochemical persistent disease (BPD) group, or structural/functional persistent disease (S/FPD) group (**Table 2**).

**Table 2:** Definition of the therapeutic response groups after initial RIT in caDTC patients.

Therapeutic response group to initial RIT	Definition ^A,B^
NED group	sup-Tg < 0.2 ng/mL or sti-Tg < 1 ng/mL and no evidence of structural/functional disease
BPD group	sup-Tg ≥ 0.2 ng/mL or sti-Tg ≥ 1 ng/mL and no evidence of structural/functional disease
S/FPD group	structural/functional evidence of disease regardless of Tg levels

A = TgAb-positive patients were excluded; B = The definition was adopted from the study by Sung et al. (2); caDTC, differentiated thyroid carcinoma in children and adolescents; RIT, radioiodine therapy; NED, no clinical evidence of disease; BPD, biochemical persistent disease; S/FPD, structural/functional persistent disease; sup-Tg, suppressed thyroglobulin; sti-Tg, stimulated serum thyroglobulin.

The therapeutic response in all three groups at the last follow-up and biochemical and structural responses in S/FPD cohort was also analyzed. In brief, the therapeutic response at the last follow-up in all three groups was classified as structural incomplete response (SIR), biochemical incomplete response (BIR), indeterminate response (IDR), or excellent response (ER) in terms of the ATA guidelines (version 2015) (9). According to the Response Evaluation Criteria in Solid Tumors 1.1 (RECIST 1.1), the assessed structural response was divided into complete response (CR), partial response (PR), stable disease (SD), and progressive disease (PD) (12, 13). The evaluation of biochemical responses was as follows: suppressed thyroglobulin (sup-Tg) < 0.2 ng/mL or a decrease in sup-Tg ≥ 25% was defined as biochemical remission (BR), the changes in sup-Tg < 25% were defined as biochemical stability (BS), and an increase in sup-Tg ≥ 25% was defined as biochemical progression (BP) (14).

The DFS in NED group was defined as time interval between initial RIT administration and the detection of disease recurrence. Progression-free survival (PFS) in BPD group was defined as time interval between initial RIT administration and biochemical disease progression or structural/functional disease recurrence. PFS in S/FPD group was defined as time interval between initial RIT and structural/functional disease progression.

### 2.5 Statistical analysis

The data are presented as medians and interquartile ranges (IQR), minimum and maximum ranges, or frequencies and percentages. DFS and PFS were evaluated using the Kaplan-Meier survival method, and the log-rank test was used to evaluate differences between groups. All tests were two-tailed. P< 0.05 was considered statistically significant.

## 3 Results

### 3.1 Baseline characteristics of caDTC patients

**Figure 1:**
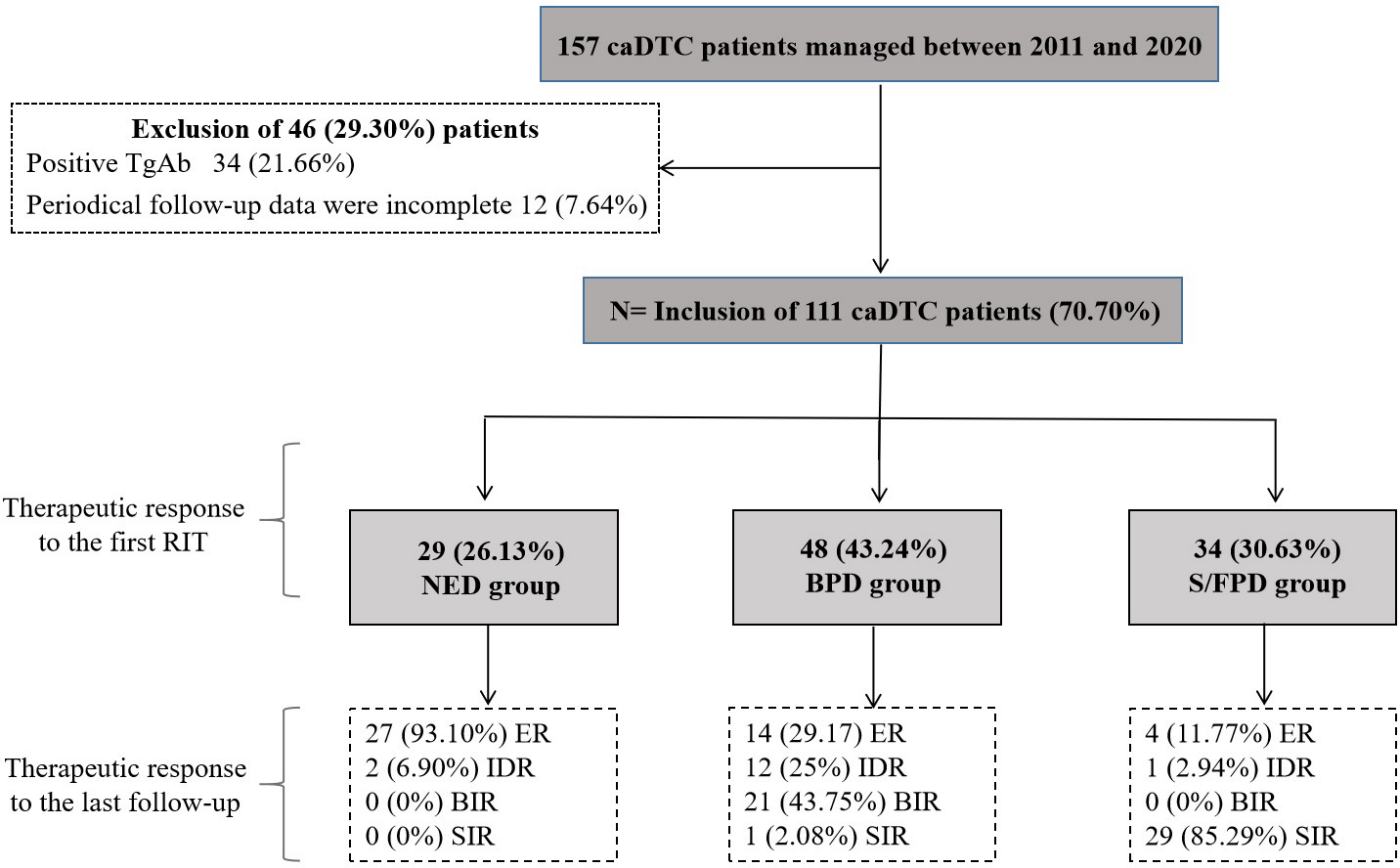
Initial caDTC patient selection and therapeutic outcomes after RIT during follow-up. RIT, radioiodine therapy; caDTC, differentiated thyroid carcinoma in children and adolescents; NED, no clinical evidence of disease; BPD, biochemical persistent disease; S/FPD, structural/functional persistent disease; ER, excellent response; IDR, indeterminate response; BIR, biochemical incomplete response; SIR, structural incomplete response.

From January 2011 to December 2020, a total of 157 caDTC patients were enrolled. After the exclusion of 46 patients (34 patients with positive TgAb and 12 patients were lost to follow-up), 70.70% (111/157) of the patients were eligible for further analyses (**Figure 1**).

The baseline clinical and pathological characteristics of 111 enrolled caDTC subjects are summarized in **Table 3**. The median age of the subjects at the first RIT was 16 years (IQR, 14-17 years), 72.1% (80 subjects) were between 15 and 18 years old at the first RIT, and the majority were female (69.4%). Of these, papillary thyroid carcinoma (PTC) was diagnosed in 107 patients (96.4%), and follicular thyroid carcinoma (FTC) was diagnosed in 4 patients (3.6%). The median primary tumor size was 2.6 cm (IQR, 1.5-3.8 cm). Regarding T staging, 45 patients (40.5%) were classified as T3-4. In addition, soft tissue invasion occurred in 45.9% of patients (51/111), and cervical lymph node metastases occurred in all patients (N1a=14; N1b =97). According to the ATA pediatric recurrence risk stratification, 61 patients (55.0%) were intermediate risk. The post-operative sti-Tg were between 2.10 and 17468.00 ng/mL (median 15.50 ng/mL). One to five post-operative RITs (median 1) were administered in 111 caDTC patients. The cumulative activity of ^131^I ranged from 50 mCi to 760 mCi (median 150 mCi). The median duration of follow-up after the first RIT was 41.32 months (range, 6.32-113.42 months).

**Table 3:** Baseline characteristics of the caDTC patients.

Age at first RIT (years)	N(%)	Median (IQR)	Range
< 15	31 (27.9)	16 (14, 17)	6-18
15-18	80 (72.1)		
Sex			
Male	34 (30.6)		
Female	77 (69.4)		
Histological type			
PTC	107 (96.4)		
FTC	4 (3.6)		
Primary tumor size (cm)		2.6 (1.5, 3.8)	0.5-5.0
Soft tissue invasion			
Yes	51 (45.9)		
No	60 (54.1)		
T stage			
T1	32 (28.8)		
T2	34 (30.7)		
T3	18 (16.2)		
T4	27 (24.3)		
N stage			
N0	0 (0)		
N1a	14 (12.6)		
N1b	97 (87.4)		
Recurrence risk			
Intermediate	61 (55.0)		
High	50 (45.0)		
Post-operative sti-Tg (ng/mL)		15.50 (5.25, 93.70)	2.10-17468.00
Cumulative RIT times		1 (1, 2)	1-5
Cumulative ^131^I activity (mCi)		150 (100, 300)	50-760
Follow-up duration (months)		41.32 (20.40, 64.14)	6.32-113.42

caDTC, children and adolescents patients with differentiated thyroid carcinoma; PTC, papillary thyroid carcinoma; FTC, follicular thyroid carcinoma; RIT, radioiodine therapy; T, tumor; N, node; sti-Tg, stimulated serum thyroglobulin.

### 3.2 Therapeutic response to the initial RIT

29 subjects (26.13%) were considered to have no clinical evidence of disease and were assigned to NED group, 48 subjects (43.24%) were identified as having biochemical persistent disease and were assigned to BPD group, and the remaining 34 subjects (30.63%) were evaluated as having structural/functional persistent disease and were assigned to S/FPD group (**Figure 1**).

### 3.3 Clinical outcomes of patients in different groups after initial RIT

#### 3.3.1 Clinical outcomes of NED patients after initial RIT

**Figure 2:**
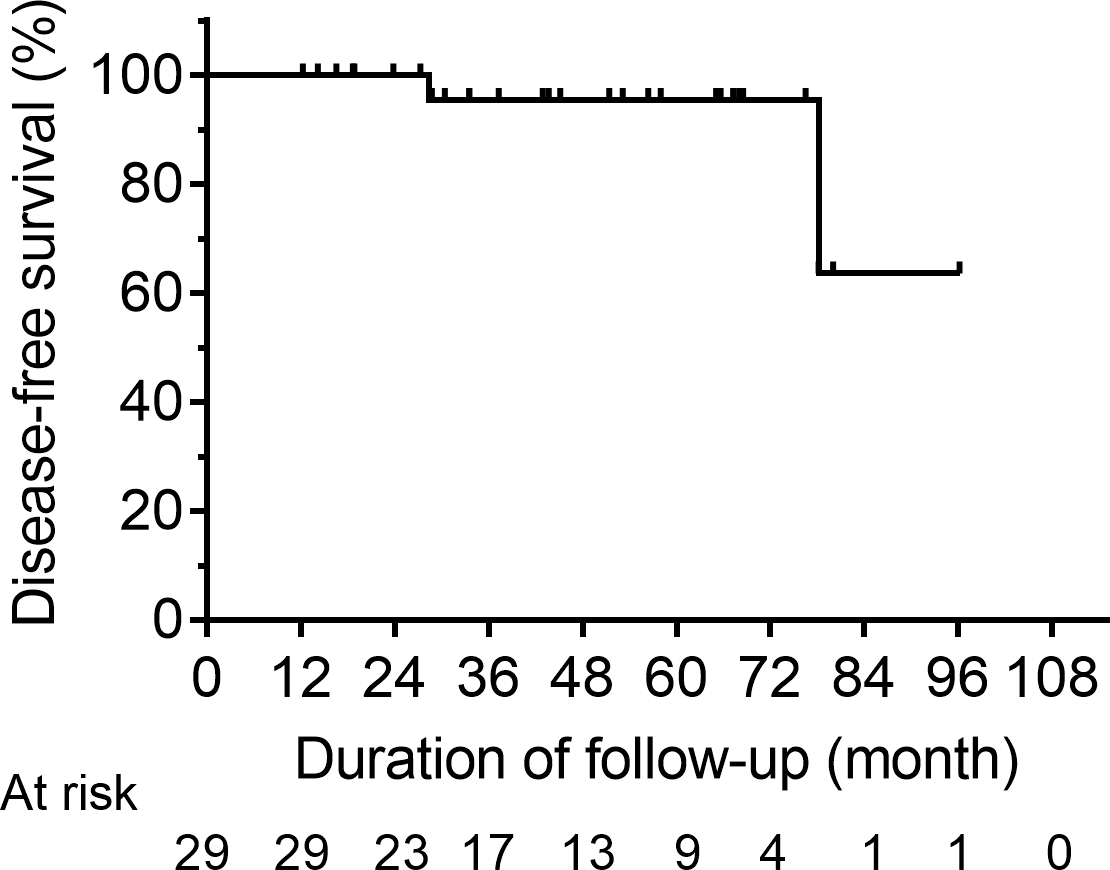
Kaplan-Meier DFS curves for the NED group. DFS, disease-free survival; NED, no clinical evidence of disease.

A total of 29 patients were assigned to NED cohort, with a median post-operative sti-Tg of 3.41 ng/mL (IQR, 2.58-5.36 ng/mL). After a median follow-up of 43.67 months (IQR, 27.82-66.43 months), 27 patients (93.10%) still had no clinical evidence of disease and remained classified as ER, 2 patients (6.90%) were reclassified as IDR, and none had BIR or SIR (**Figure 1**). Kaplan-Meier survival analysis of DFS in NED group is shown in **Figure 2**. The median DFS was not observed. The 1-year, 3-year, and 5-year DFS rates of patients in NED group were 100%, 95.5%, and 95.5%, respectively.

#### 3.3.2 Clinical outcomes of BPD patients after initial RIT

**Figure 3:**
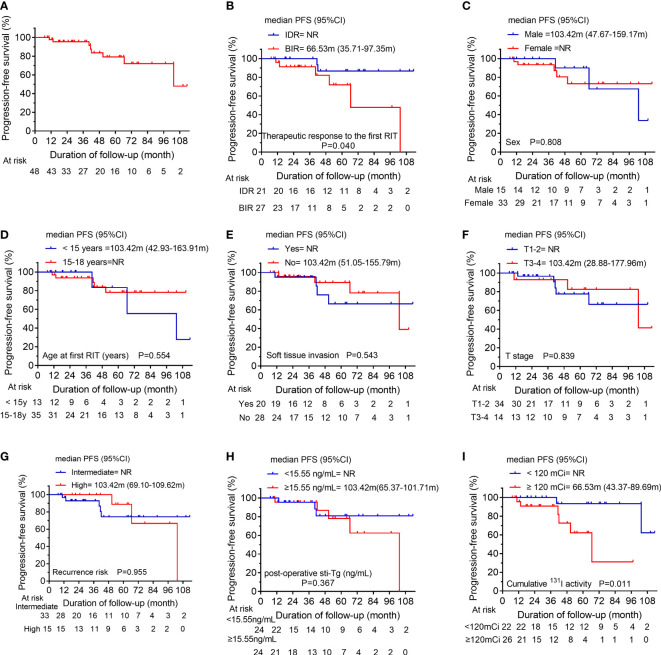
**(A)** Kaplan-Meier progression-free survival (PFS) curves for the BPD group. Comparison of PFS curves for the BPD group according to **(B)** the therapeutic response to the first RIT, **(C)** sex, **(D)** the age at first RIT, **(E)** soft tissue invasion, **(F)** T stage, **(G)** the recurrence risk, **(H)** post-operative sti-Tg, and **(I)** the cumulative ^131^I activity. BPD, biochemical persistent disease; RIT, radioiodine therapy; T, tumor; sti-Tg, stimulated serum thyroglobulin; NR, not reached.

After the initial RIT, IDR was observed in 21 patients, BIR was observed in 27 patients and these patients were assigned to BPD cohort (n=48), with a median post-operative sti-Tg of 15.55 ng/mL (IQR, 9.39-28.71 ng/mL). Of these, the median cumulative activity of ^131^I was 120 mCi (IQR, 88.75-270 mCi). After a median follow-up of 40.85 months (IQR, 19.24-65.66 months), 14 patients (29.17%) achieved ER. In contrast, 34 patients (70.83%) had persistent/recurrent disease at the last follow-up; the majority had BIR (n=21; 43.75%), 25.00% (n=12) had IDR, and only 2.08% (n=1) had SIR (**Figure 1**). In the analysis of PFS, 8 patients had disease progression. The median PFS of BPD group was 103.42 months (**Figure 3A**). Then, the 1-year, 3-year, and 5-year PFS rates of patients in BPD group were 97.7%, 95.3%, and 79.2%, respectively. Moreover, subgroup analysis revealed significant differences between the subgroups in terms of the therapeutic response to the first RIT (**Figure 3B**) and the cumulative ^131^I activity (**Figure 3I**), while there were no significant differences between subgroups based on sex, age at first RIT, soft tissue invasion, T stage, recurrence risk, or post-operative sti-Tg (**Figures 3C–H**).

#### 3.3.3 Clinical outcomes of S/FPD patients after initial RIT

A total of 34 patients were assigned to S/FPD cohort, with a median post-operative sti-Tg of 149.80 ng/mL (IQR, 54.13-618.33 ng/mL). At a median follow-up of 33.75 months (IQR, 18.84-53.70 months), ER, IDR, BIR, and SIR were achieved in 4 (11.77%), 1 (2.94%), 0 (0%), and 29 (85.29%) patients, respectively (**Figure 1**). Overall, patients in S/FPD cohort received a median of 2 RITs (IQR, 2-3) with a median cumulative ^131^I activity of 340 mCi (IQR, 215-422.5 mCi).

The biochemical and structural responses in S/FPD cohort are shown in **Table 4**. For biochemical evaluation, 17 patients (50.00%) achieved BR, 7 patients (20.59%) achieved BS, and 10 patients (29.41%) achieved BP. Two patients had no definite structural lesions and were classified as having functional persistent disease based on ^131^I WBS. After the exclusion of 2 patients without definite structural lesions, the remaining 32 patients had definite structural lesions and were evaluated for structural response. Of whom, CR, PR, SD and PD, were achieved in 3 (9.38%), 8 (25.00%), 11 (34.37%) and 10 (31.25%) patients, respectively. Then, the disease control rate (DCR) was 68.75% (22/32) and the objective response rate (ORR) was 34.38% (11/32).

**Table 4:** Biochemical and structural responses in the S/FPD cohort.

Biochemical response (n=34)		Structural response (n=32)	
BR	17 (50.00%)	CR	3 (9.38%)
BS	7 (20.59%)	PR	8 (25.00%)
BP	10 (29.41%)	SD	11 (34.37%)
BBR	24 (70.59%)	PD	10 (31.25%)
		ORR	11 (34.38%)
		DCR	22 (68.75%)

S/FPD, structural/functional persistent disease; BR, biochemical remission; BS, biochemical stability; BP, biochemical progression; BBR, biochemical benefit rate, BR+BS; CR, complete response; PR, partial response; SD, stable disease; PD, progressive disease; ORR, objective response rate = CR+PR; DCR, disease control rate, CR+PR+SD.

**Figure 4:**
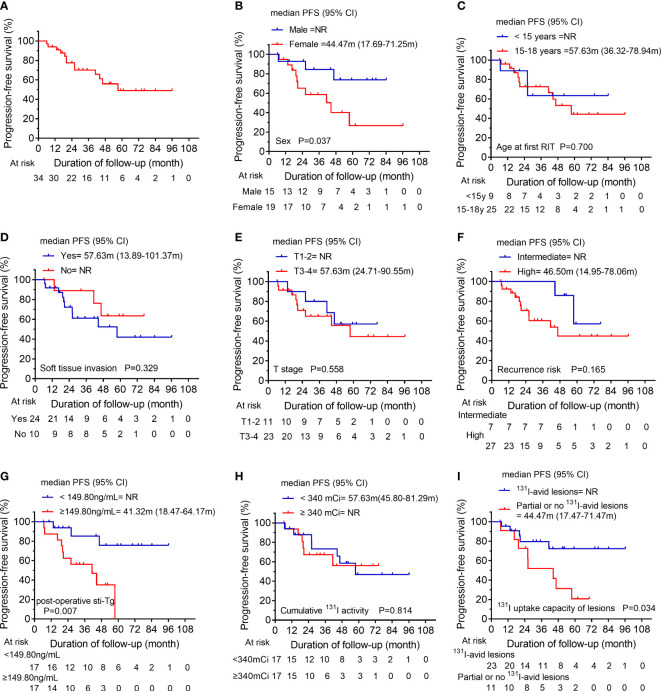
**(A)** Kaplan-Meier progression-free survival (PFS) curves for the S/FPD group. Comparison of PFS curves for the S/FPD group according to **(B)** sex, **(C)** age at the first RIT, **(D)** soft tissue invasion, **(E)** T stage, **(F)** recurrence risk, **(G)** post-operative sti-Tg, **(H)** cumulative ^131^I activity and **(I)** ^131^I uptake capacity of lesions. S/FPD, structural/functional persistent disease; RIT, radioiodine therapy; T, tumor; sti-Tg, stimulated serum thyroglobulin; NR, not reached.

In analysis of PFS, 13 patients had disease progression during follow-up. The median PFS of S/FPD group was 57.63 months (**Figure 4A**). Then, the 1-year, 3-year, and 5-year PFS rates of patients in S/FPD group were 93.9%, 70.0%, and 48.6%, respectively. Poorer PFS was found in patients who were female (**Figure 4B**), those who had post-operative sti-Tg higher than 149.80 ng/mL (**Figure 4G**), and those who had partially or no ^131^I-avid lesions (**Figure 4I**). Nevertheless, no statistically significant differences were found between subgroups in terms of age at the first RIT, soft tissue invasion, T stage, recurrence risk, or cumulative ^131^I activity (**Figures 4C–F, H**).

## 4 Discussion

caDTC has a high recurrence rate but poses a low risk of death, and patients diagnosed with caDTC typically present with painless neck masses, with only a few patients presenting with hoarseness, dyspnea, and dysphagia (11, 15, 16). To the best of our knowledge, some studies have focused on the dynamic therapeutic response; however, the overall number of caDTC patients was small, and these studies did not focus on the relationship between prognosis and the patient’s pathological characteristics at initial treatment (2, 3). Due to the particularity of children and adolescents, higher evidence-based medical evidence of RIT is urgently needed for caDTC patients. The retrospective study systematically investigated the clinical and follow-up data of caDTC patients who received RIT in two tertiary Grade A hospitals in China. Notably, we divided 111 caDTC patients into the NED cohort, BPD cohort and S/FPD cohort according to their serology and medical imaging from 6-12 months after initial RIT, and the long-term objective prognosis of the above three cohorts was also explored.

Biko et al. (17) found that the prognosis of caDTC is excellent, with a 10-year overall survival (OS) of 98%. As expected, we observed that caDTC patients in NED cohort displayed a favorable prognosis after initial RIT, and approximately 93.10% of patients maintained ER during the long-term follow-up. Interestingly, the median DFS value was not observed, and the 5-year DFS rate of patients in NED group was 95.5%. In a cohort of 63 caDTC patients who underwent total thyroidectomy and received RIT, Zanella et al. (3) found that 90% of patients (19/21) with ER after initial treatment remained disease-free after a median follow-up of 6.0 (2.7-10.0) years. Taken together, these results combined with those of present study consistently indicate that the general prognosis of patients in NED cohort is good.

In the present study, the BPD cohort consisted of 21 IDR patients and 27 BIR patients. After a median follow-up of 40.85 months, only 29.17% of patients achieved ER. Furthermore, we found that 8 patients had disease progression, with a 5-year PFS rate of 79.2% in BPD cohort. Additionally, the clinical factors associated with PFS in BPD cohort were assessed, and it was observed that patients with BIR in the first evaluation and/or a cumulative ^131^I activity ≥ 120 mCi might have a poorer PFS than other patients. Thus, we suggest that more active follow-up may be needed for patients who have BIR after the initial RIT or a cumulative ^131^I activity greater than 120 mCi. However, other clinical factors such as sex, soft tissue invasion and so on, were not associated with PFS.

RIT has become the first-line therapeutic strategy for treating residual thyroid carcinoma or metastatic lesion, and improving PFS, as well as OS, in patients with ^131^I-avid lesions (9, 18, 19). In the present study, only 30.63% of patients were classified as S/FPD cohort, and the corresponding biochemical and structural assessments were performed. After receiving a median cumulative dose of 340 mCi of RIT, 70.59% of patients had biochemical benefits, and 68.75% of patients achieved structural disease control (**Table 4**).

Of note, we found that the median PFS of S/FPD group was 57.63 months, with a 5-year PFS rate of 48.6%. Additionally, we further explored the factors that might influence the PFS in S/FPD cohort and found that male children, ^131^I-avid lesions, and post-operative sti-Tg < 149.80 ng/ml were associated with longer PFS, and unrelated factors included T stage, cumulative ^131^I activity and recurrence risk stratification.

The disparities in adult DTC between sexes have been well documented. Adult females have been found to have a higher incidence of DTC than adult males; however, adult males appear to have worse clinical outcomes (20, 21). Rajoria et al. (22) reported that estrogen might have a certain protective effect on DTC, but the definitive mechanism is not well understood. In addition, Zhang et al. demonstrated that testosterone could promote thyroid cancer progression in a transgenic mouse model of FTC (23). In contrast, in our study, we found that male patients had a longer PFS than female patients in S/FPD group. In a retrospective study involving 118 caDTC patients, Liu et al. (1) demonstrated that sex had no significant influence on patients’ long-term clinical outcomes. Therefore, sex may have different effects on the clinical prognosis of DTC in children and adults, and more high-quality studies are needed to verify this hypothesis in the future.

Durante et al. (24) performed a retrospective study involving 444 patients with metastatic DTC and showed that the 10-year OS rate in patients with no ^131^I-avid lesions was significantly lower than that of patients with ^131^I-avid lesions (10% vs. 56%). Thus, RIT is highly effective in a selected group of patients with ^131^I-avid metastatic lesions. The long-term survival of caDTC patients remains satisfactory despite its high recurrence and metastasis rates after surgery. In the current study, the OS was not reached due to our short follow-up. However, we found that ^131^I-avidity of the lesion was a reliable predictor for longer PFS in caDTC patients. The reason might be that patients with partially or no ^131^I-avid lesions were ineligible for RIT or unable to reach the absorbed dose required to control disease progression.

sti-Tg is a reliable biochemical marker for reflecting the tumor burden during follow-up in the post-operative management of caDTC patients (1, 11). Moreover, post-operative sti-Tg has been suggested as a prognostic factor in caDTC patients by several studies (25–28). A retrospective study of 118 caDTC patients in China indicated that a post-operative sti-Tg higher than 17.8 ng/mL was an independent predictor of persistent/recurrent disease (1). Zanella et al. (26) studied 17 caDTC patients, and a post-operative sti-Tg < 37.8 ng/mL was found to predict disease‐free status with high sensitivity and specificity. Moreover, a multicenter real-world study in Italy showed that the post-operative sti-Tg≥27.2 ng/ml was significantly correlated with non-ER 1-year after RIT. Besides, the post-operative sti-Tg and 1-year treatment response categories after RIT had the capability of predicting the last disease status in pediatric caDTC patients (29). Notably, at a median follow-up of 33.75 months, we found that the PFS of S/FPD patients with post-operative sti-Tg ≥149.80 ng/mL was significantly shorter than that of patients with post-operative sti-Tg <149.80 ng/mL. This phenomenon further confirmed that post-operative sti-Tg was related to the prognosis of RIT in the S/FPD group. Our findings might help guide the follow-up of caDTC patients with S/FPD to distinguish those who need more aggressive treatment and closer follow-up.

Instead, our analysis suggests that the risk stratification of ATA class does not possess a predictive function for PFS in BPD or S/FPD groups, and this finding aligns with the evidence presented by other studies (3, 29).

Nevertheless, the present study had several limitations. First, due to the rarity of the caDTC patient population, the small sample size of this study may have caused unavoidable selection bias. Second, TgAb-positive patients were not included in this study due to the controversy regarding disease status assessment and serological efficacy evaluation. Third, molecular characteristics were not included in the prognostic analysis because there was a failure to identify the molecular characteristics of all patients. Fourth, the heterogeneity of the management and follow-up approaches, and its retrospective design. Thus, a multicenter, larger cohort-based study from China is needed to confirm the importance of RIT for the long-term outcomes of caDTC patients.

## 5 Conclusions

In conclusion, the response to initial RIT could be helpful for defining subsequent treatment and follow-up strategies for caDTC patients. Our study showed that post-operative sti-Tg and ^131^I-avidity of lesions are correlated with PFS.

## Data availability statement

The original contributions presented in the study are included in the article/supplementary material. Further inquiries can be directed to the corresponding authors.

## Ethics statement

The studies involving human participants were reviewed and approved by Ethical Committee of the Affiliated Hospital of Qingdao University (Ethics approval number: QYFYWZLL27344). Written informed consent to participate in this study was provided by the participants’ legal guardian/next of kin.

## Author contributions

CW, XW, and RW contributed to the conception and design of the study. GL, GW, CL, QZ, and JL assisted with data acquisition. CW and YL conducted the statistical analyses and drafted the manuscript. XL, QL, and MS critically revised the manuscript. CW, GL, and YL contributed equally to this work. All authors contributed to the article and approved the submitted version.
